# HIV transmitted/founder vaccines elicit autologous tier 2 neutralizing antibodies for the CD4 binding site

**DOI:** 10.1371/journal.pone.0177863

**Published:** 2017-10-11

**Authors:** Nathanael P. McCurley, Arban Domi, Rahul Basu, Kevin O. Saunders, Celia C. LaBranche, David C. Montefiori, Barton F. Haynes, Harriet L. Robinson

**Affiliations:** 1 GeoVax Inc., Smyrna, GA, United States of America; 2 Duke Human Vaccine Institute, Durham, NC, United States of America; University of Toronto, CANADA

## Abstract

Here we report the construction, antigenicity and initial immunogenicity testing of DNA and modified vaccinia Ankara (MVA) vaccines expressing virus-like particles (VLPs) displaying sequential clade C Envelopes (Envs) that co-evolved with the elicitation of broadly neutralizing antibodies (bnAbs) to the CD4 binding site (CD4bs) in HIV-infected individual CH0505. The VLP-displayed Envs showed reactivity for conformational epitopes displayed on the receptor-binding form of Env. Two inoculations of the DNA-T/F vaccine, followed by 3 inoculations of the MVA-T/F vaccine and a final inoculation of the MVA-T/F plus a gp120-T/F protein vaccine elicited nAb to the T/F virus in 2 of 4 rhesus macaques (ID50 of ~175 and ~30). Neutralizing Ab plateaued at 100% neutralization and mapped to the CD4bs like the bnAbs elicited in CH0505. The nAb did not have breadth for other tier 2 viruses. Immunizations with T/F followed by directed-lineage vaccines, both with and without co-delivery of directed-lineage gp120 boosts, failed to elicit tier 2 neutralizing Ab for the CD4bs. Thus, pulsed exposures to DNA and MVA-expressed VLPs plus gp120 protein of a T/F Env can induce autologous tier 2 nAbs to the CD4bs.

## Introduction

A challenge for HIV vaccine development is the generation of neutralizing antibody for the diversity of primary isolates capable of mediating transmission. Recombinant antibodies with broad neutralizing potential (bnAbs) have been isolated for multiple specificities from humans with natural HIV infections [1, 2]. Most bnAbs have atypical characteristics including high levels of somatic mutations, long third complementarity-determining regions of the heavy chain and polyreactivity for non-HIV antigens [3–5]. Analyses of bnAbs for the same epitope, but from different patients, suggest that specificities for bnAb are generated by serial mutations of unmutated common ancestors (UCA)[6–8]. In this study, we use a clade C lineage from a South African individual (CH0505) followed from the time of infection to the development of bnAb to the CD4bs to construct a directed-lineage vaccine to test whether vaccination can replicate the generation of either broad or autologous nAbs to the CD4bs that occurred in the CH0505 infection [9, 10]. In bnAb lineages in the CH505 individual, the ability of lineage member antibodies to mediate autologous neutralization preceded the ability to broadly neutralize heterologous HIV isolates (9,10).

The clade C infection in CH0505 was the first characterized for the co-evolution of Env and Ab during the generation of bnAbs to the CD4bs [9]. An important feature for using this lineage of Envs was the ability of the transmitted/founder (T/F) Env to bind to a V_H_4-59 UCA for bnAb to the CD4bs (11). A second important feature for aiming to elicit bnAbs with CH0505 Envs was that the bnAb developed during natural infection with relatively few mutations in the V_H_ of the UCA (~ 15%) and within a relatively short time span (~2 years).

Here we report on the construction, antigenicity and initial immunogenicity testing of vaccines displaying the native forms of the CH0505 T/F, week 53, week 78 and week 100 Envs on Virus-Like-Particles (VLPs). Our hypothesis was that trimeric CH0505 Envs displayed on virus-like particles (VLPs), with or without co-administered gp120, would elicit bnAb by presenting the same angle of approach to the CD4bs as the virion Envs that co-evolved with the generation of bnAb to the CD4bs in the infected individual CH0505.

## Materials and methods

### Cells

293T cells (ATCC CRL-3216), a human embryonic kidney cell line immortalized by the SV40 T-antigen, and DF-1 cells (ATCC CRL-12203), a spontaneous line of chicken embryo fibroblasts derived from endogenous Avian leucosis virus-free chickens, were obtained from ATCC. TZM-Bl cells, an indicator cell line for HIV infection, was obtained from the NIH AIDS Reagent Program (catalog # 8129). Specific pathogen free chicken embryo fibroblasts (CEF) were obtained from Charles River Laboratories International Inc. 293T cells and DF-1 cells were maintained in DMEM medium (Corning) supplemented with 10% fetal bovine serum (FBS, Gibco), penicillin (100 IU/mL) and streptomycin (100 μg/mL) (Gibco). For infections, transfections, and cell culture following these procedures, DMEM (Corning) supplemented with 2% FBS was used. CEF were maintained in EMEM (BioWhittaker) supplemented with 2.5% FBS (Hyclone), streptomycin, neomycin (Sigma), and L-glutamine (BioWhittaker).

### Construction of DNA and MVA vaccines

A DNA vaccine was constructed for the T/F virus (DNA-T/F) while MVA vaccines were constructed for the T/F, week 53.16, week 78.33 and week 100.B6 Envs. Both the DNA and MVA vaccines were constructed using certified reagents in a dedicated room. Construction of the DNA vaccine used synthetic DNA sequences whereas the construction of the MVA vaccines used synthetic shuttle vectors and a parental MVA that had been harvested in 1974 before the appearance of bovine spongiform encephalopathy (BSE) and sent in 2001 to Dr. Bernard Moss at NIAID, where it was plaque purified 3 times using certified reagents from sources free of BSE. Vaccines were constructed using standard techniques.

**Fig 1:**
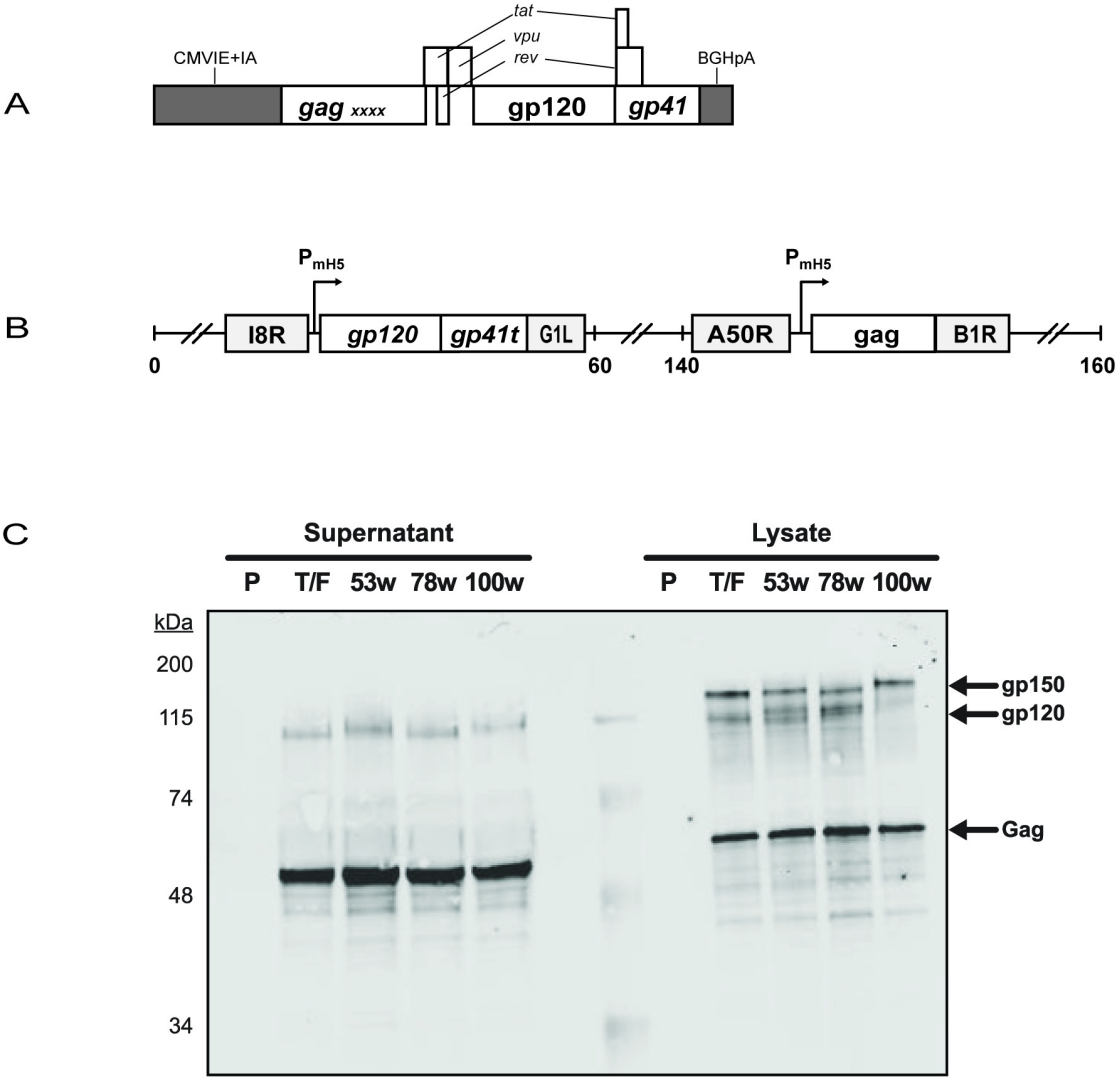
DNA and MVA constructs and their expression. (A) Schematic for the expression cassette of the DNA vaccine expressing 505 T/F sequences. CMVIE+IA, CMV-immediate early promoter plus intron A; BGHpA, bovine growth hormone polyadenylations sequences; *gp120* and *gp41*, HIV sequences encoding the receptor binding and transmembrane subunits of Env; *gag*, HIV sequences encoding the group-specific antigens of HIV; X, inactivating point mutations in the zinc fingers for packaging of HIV RNA. (B) Schematic of MVA expressing MVA-T/F sequences. I8R and G1L, conserved vaccinia sequences flanking the insertion site for the T/F *env;* A50R and B1R, conserved sequences flanking the insertion site for the T/F *gag;* P_mH5_, a modified immediate early H5 vaccinia promoter; *gp41t*, gp41 truncated at amino acid 36 of the endodomain. Numbers indicate positions in the MVA genome which is abbreviated and not to scale. (C) Western blot of MVA-expressed Gag and Env proteins. Supernatants and cell lysates were collected from 293T cells infected with the indicated recombinant MVA vaccines or the parental MVA (P), separated by SDS-PAGE, and western blotted for detection of Gag and Env.

The DNA vaccine used the pGA1 expression vector to express Gag, Tat, Rev, Vpu and Env by subgenomic splicing of a single RNA[11] (Fig 1A). Packaging of viral RNA was minimized by the deletion of packaging sequences found in the 5’ untranslated region and by inactivating point mutations in the two zinc fingers in Gag as previously described [11]. The DNA vaccine was designated DNA-T/F.

The MVA vaccines were constructed using shuttle vectors. The pLW76 shuttle vector (Wyatt and Moss, unpublished data) was used to place T/F *gag* sequences in a modified and restructured insertion site III and the pLW73 shuttle vector to insert *env* sequences between two essential vaccinia genes (I8R and G1L) [12] (Fig 1B). A T/F Gag recombinant MVA was recombined with serial *env* shuttle vectors to generate the lineage vaccine. *Env* sequences were truncated for nucleotides encoding the 115 C-terminal amino acids of the endodomain of gp41 to eliminate three previously identified endocytic sequences within gp41 that reduce display of Env on VLPs and the plasma membranes of infected cells [13]. *Gag* and *Env* inserts were optimized for the codon usage of vaccinia virus and sequences encoding termination of vaccinia transcripts were eliminated by using alternate codons [12]. The shuttle vectors used the modified H5 early/late promoter to drive transcription [14]. The pLW73 Env expression cassette was modified to include a proprietary upstream ATG that reduces Env expression to levels that allow better processing of the overexpressed Env as demonstrated by more complete proteolytic cleavage of the gp150 precursor into gp120 and gp30 subunits[15]. The MVA vaccines were designated MVA-T/F, MVA53C, MVA78C, and MVA100C.

### Transfection and infections

Transfections were carried out using Lipofectamine 2000 (Invitrogen) using the manufacturer’s directions. Infections were carried out on sub confluent monolayers using purified stocks of the MVA vaccines at a MOI of approximately 1.

### Immunostaining

Co-expression of both Gag and Env in MVA vectors was verified in infected DF1 cells using immunostaining for Gag, Env, and MVA and standard techniques. H12.5C mouse monoclonal antibody (NIH AIDS Repository, #3537) was used to stain for Gag, ID6 mouse monoclonal (NIH AIDS Repository, #2343) for Env, and anti-WR a rabbit polyclonal Ab (kindly supplied by Bernard Moss) for MVA.

### Western blots

Western blots were conducted on supernatants and lysates of transiently transfected 293T cells or on infected CEFs or DF-1 cells. H12.5C was used as the primary Ab for Gag and ID6 as the primary Ab for Env. Samples, separated by SDS-PAGE at various percentages, were transferred to nitrocellulose membranes for staining using standard western blotting techniques.

### Immuno-electron microscopy

Cells were plated on poly-D-lysine-treated Aclar discs (Electron Microscopy Sciences) in 12-well tissue culture plates. For expression of VLPs by DNA, 293T cells were transfected with 0.5 μg of DNA-T/F. For expression of VLPs by the MVA vaccines, DF-1 cells were infected at a MOI of 1 with MVA-T/F, MVA53C, MVA78C, or MVA100C. Two-days later, vaccine-expressing cells were incubated for 2 hours at 37^o^ with a mixture of 10 μg/ml of trimer-specific recombinant Abs PGT 145 (specific to V1V2 tip) and PGT 151 (specific to the cleaved gp120-gp41 interface) and then washed several times with culture medium. Cells were then fixed with 1% glutaraldehyde in 0.1 M phosphate buffer (pH7.4). Immediately after the cells were placed in fixative, they were delivered to the Emory University Robert P. Apkarian Integrated Electron Microscopy Core for incubation with 6 nm colloidal gold particle conjugated goat anti-human secondary antibody. Upon finishing antibody incubation, cells were further fixed with 2.5% glutaraldehyde, and post fixed with 1% osmium tetroxide. Cells were then dehydrated with increasing percent solutions of ethanol and embedded in Eponate 12 resin. Ultrathin sections were cut at 70–80 nm thicknesses, and stained with 5% uranyl acetate and 2% lead citrate. Cell imaging was done on a JEOL JEM-1400 transmission electron microscope (JEOL Ltd) equipped with a Gatan US1000 CCD camera (Gatan).

### Antigenicity studies

Monoclonal and recombinant human Abs used for antigenicity mapping included PG9, VRC01 and F240 obtained from the AIDS Reagent program (catalog #12149, #12033, and #7623 respectively); PGT 145, PGT 121, PGT 135, and PGT 151 obtained from the International AIDS Vaccine Initiative Antibody Consortium; and 17B obtained from Dr. James Robinson, Tulane University. For antigenicity studies, DNA-transfected, or MVA-infected, 293T cells were gently pipetted from the plate into a single-cell suspension and incubated for 10 minutes on ice in the presence or absence of 3μM sCD4 (NIH AIDS Reagent Program, catalog #7356). Cells were immediately surface stained (without washing) for Env with 0, 0.5, or 5 μg/mL of the test antibodies, washed, and stained with the secondary Ab, PE-labeled anti-human IgG (Santa Cruz Biotechnology). After washing, cells were fixed in Cytofix/Cytoperm (BD Biosciences) and stained intracellularly for Gag with FITC-labeled KC57 (Beckman Coulter). Staining with anti-Env and anti-Gag Abs was performed for 30 minutes on ice. Flow cytometric analyses collected a minimum of 10,000 live cells using a FACScanto. Live cells were gated on Gag, and then mean fluorescence intensity (MFI) was measured for each anti-Env antibody concentration in the presence and absence of sCD4. The MFI data-set for each Ab was normalized to its highest value, which was set as 100%.

### Animal study

The immunogenicity study was conducted at the New Iberia Research Center, Lafayette, Louisiana in accordance with all rules of the American Association for Laboratory Animal care and under the supervision of the Institutional Animal Clinical Care and Use Committee. Six male and six female 4–6 year-old Indian origin Rhesus macaques, purpose bred on site at New Iberia Research Center, were socially housed in side by side wall hung Group 3 and/or Group 4 enrichment cages based on current body weights and body stature of each NHP. NHPs were offered commercially available Monkey Chow with rations calculated based on animal size and age. Fresh fruits and vegetables were offered daily and novel treats (such as seed, nuts and other on site prepared foods) were offered to encourage species specific foraging activities. Durable manipulable objects such as plastic balls, cone toys, and chew toys were part of an enriched cage for all NHPs. Smaller durable toys, such as rattles, beads on chains and mirrors were attached to cages and perches were available in all housing units. Animals were evaluated daily by research staff for signs of illness, distress, injury and/or variances from species and/or animal specific normal behaviors such as inappetence, variance in activity levels, guarding, etc. One animal died unexpectedly during the course of the study. The findings on necropsy supported the cause of death as acute gastrointestinal dilation. NHPs were sedated for vaccine administration and sample collections (blood and rectal fluids) by Intramuscular administration of Ketamine (5–10 mg/kg) and/or Telazol (4–6 mg/kg) based on last body weight on file to maintain an appropriate plane of sedation for safe handling. Food was removed a minimum of 2 hours prior to scheduled sedation. After the experiment, based on antibody titer, some animals were continued in the study and some were returned to group social housing in the NIRC RHM colony (for future potential use on other research programs).

Animals were randomized into 3 gender-balanced study groups of 4 animals each (Table 1). For inoculations, 3 mg of DNA-T/F in 1 ml of PBS with 1%(v/v) ethanol, pH 7.5 was inoculated by needle and syringe into the left thigh, 1x10^8^ TCID_50_ of MVA in 1 ml of PBS with 7.3% (w/v) sucrose, pH 7.3, into the right thigh and 300 μg of gp120 in 600 μg of alhydrogel (Brenntag Biosector, CAS 21645-51-2) in PBS, pH 7, into the left thigh. All groups were primed two times with DNA T/F. Group 1 was then boosted three times with MVA-T/F and received a final immunization with MVA T/F + gp120T/F. Group 2 was boosted sequentially with MVA-T/F, MVA53C, and MVA78C, and received a final immunization with MVA100C plus gp120-100C. Group 3 received gp120 protein boosts for the directed-lineage starting at treatment 4 (boost 2), with the gp120 sequences being from the Env used in the preceding MVA inoculation. Immunizations were at eight week intervals until the 5th and 6th treatments which were at 16 week intervals. Sera, PBMC, and rectal swabs were collected at regular intervals throughout the trial. Depending on Ab titers, some animals were continued in the trial, whereas others were returned to social housing at the NIRC (for use in other potential trials). The general health, weights, clinical blood counts (CBC) and clinical chemistries of animals were monitored throughout the trial.

**Table 1:** Immunization schema^1^.

Group (n = 4)	T #1 (t = 0)	T #2 (t = 8w)	T #3 (t = 16w)	T #4 (t = 24w)	T #5 (t = 40w)	T#6 (t = 56w)
1 (T/F)	DNA-T/F (left)	DNA-T/F (left)	MVA-T/F (right)	MVA-T/F (right)	MVA-T/F (right)	MVA-T/F (right)
						gp120-T/F (left)
2 (D/L)	DNA-T/F (left)	DNA-T/F (left)	MVA-T/F (right)	MVA53C (right)	MVA78C (right)	MVA100C (right)
						gp120-100C (left)
3 (D/L+ gp120)	DNA-T/F (left)	DNA-T/F (left)	MVA-T/F (right)	MVA53C (right)	MVA78C (right)	MVA100C (right)
				gp120-T/F (left)	gp120-53C left)	gp120-78C (left)

^1^DNA (3 mg), MVA (1X10^8^ TCID_50_) and gp120 protein (0.3 mg of protein in 0.6 mg of Alhydrogel) were delivered in 1 ml intramuscularly to the indicated thigh using a hypodermic needle and syringe.

### Assays for Ab

Binding Ab titers to CH0505 gp120-T/F, resurfaced core 3 (RSC3) (specific for Ab to the CD4bs), and RSC3Δ371I (RSC3 mutationally inactivated for binding of Ab to the CD4bs) were assessed by Enzyme-linked-immunosorbent–assays (ELISA). CH0505 gp120 was produced in 293F cells at the Duke Human Vaccine Institute, and RSC3 and RSC3Δ371I were obtained from the DAIDS AIDS Reagent Repository [16, 17]. Assays included a standard curve of macaque IgG captured by goat anti-rhesus Ab and results interpolated to estimate μg of specific Ab per ml of serum as previously described [18].

Elicited sera were tested for neutralizing activity on TZM-bl cells using pseudovirions for the CH0505 lineage Envs (all tier 2) plus the Env of a tier 1 virus that appeared at week 4.3 post infection[9, 19]. Sera that neutralized the T/F pseudovirion were epitope mapped using two mutant viruses that inhibit neutralizing Ab for V1V2 on the CH0505 Env (N160A and N160A.N173A)[20]; two that inhibit neutralizing Ab for the CD4bs (N280D and G458Y)[21] and two that inhibit V3 glycan neutralization (N301A and N334A)[22]. Sera were also tested for CD4bs precursor activity using the CH0505TF.gly3.276 and CH0505TF.gly4 mutants that are highly sensitive to bnAb precursors for the CH0505 lineage[23], and 426c.DM and 426c.TM mutants grown in 293S/GnTI cells that are highly sensitive to VRC01-like bnAb precursors[24].

## Results

### Expression of the DNA and MVA vaccines

The DNA-T/F, MVA-T/F, MVA53C, MVA78C, and MVA100C vaccines were characterized for expression of Gag and Env proteins using western blots on 48 hour transfected (DNA vaccine) or infected (MVA vaccines) 293T cells (Fig 1C and data not shown). For both DNA and MVA vaccines, Gag, which was expressed in the absence of protease, was present as uncleaved pr55 in both cell lysates and supernatants. Env was present as uncleaved gp160 (DNA vaccine), or gp150 (MVA vaccines) as well as cleaved gp120 forms in cell lysates and almost exclusively as the mature gp120 form in supernatants. Consistent with the formation of budding VLPs, expressed protein moved from being primarily detected in cell lysates to being predominantly detected in cell supernatants (data not shown).

**Fig 2:**
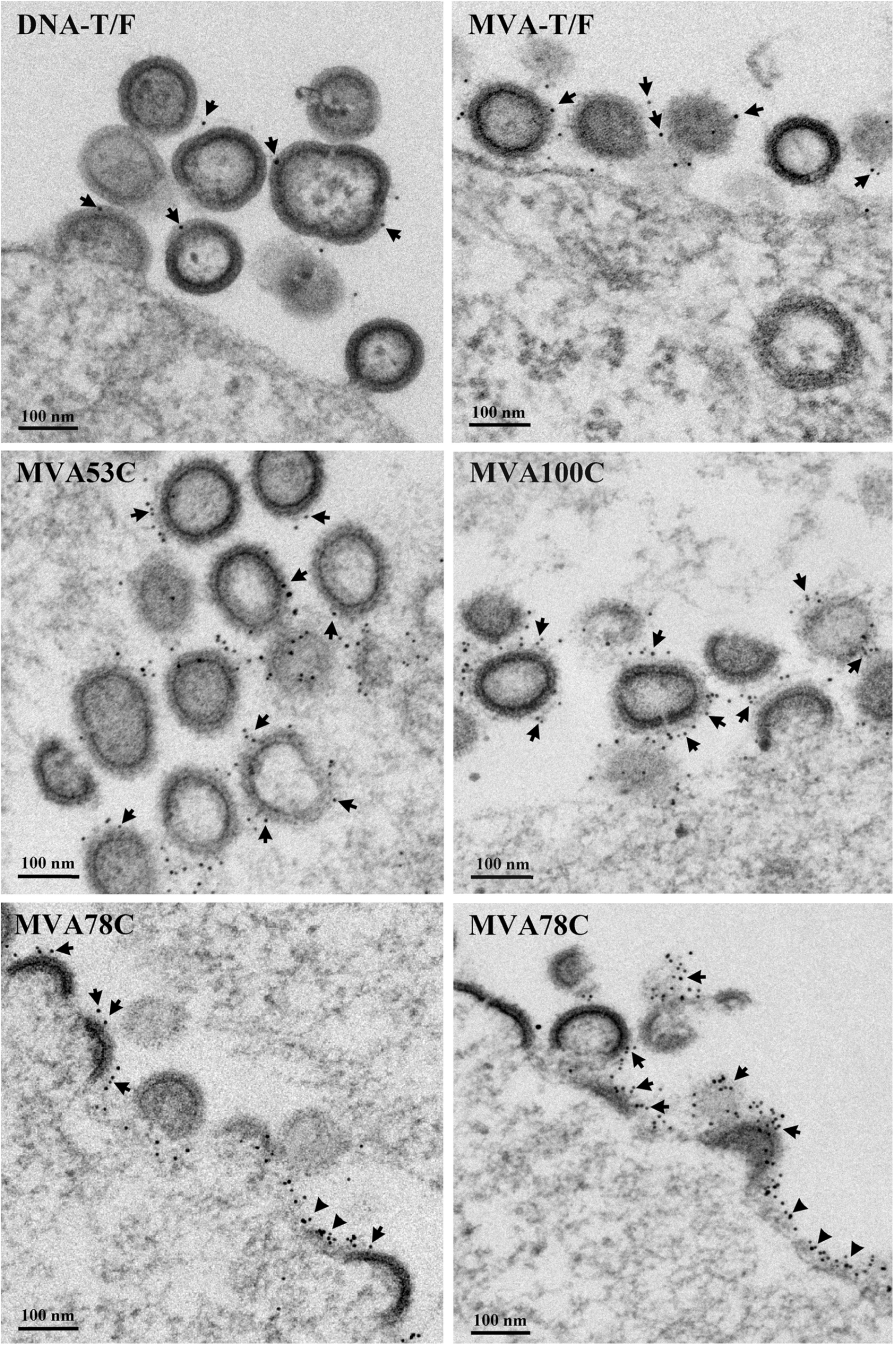
Electron micrographs of VLPs expressed by the DNA and MVA vaccines. Thin section electron micrographs were immunogold stained for Env using the PGT145 and PGT151 Ab that bind native trimers (see methods). The DNA vaccine is expressed in transiently transfected 293T cells and the MVA vaccine in infected DF1 cells. The VLPs being analyzed and nanometer (nm) size markers are indicated in the panels. Arrows, indicate examples of immunogold staining on VLPs. The triangles point to examples of immunogold staining on the plasma membranes of vaccine infected cells.

Visualization of the VLPs produced by the DNA and MVA vaccines was undertaken using thin-section electron microscopy and immunogold staining with trimer (PGT 145) and cleavage-specific (PGT 151) Ab for Env (Fig 2). Env display was observed on released VLPs, budding VLPs and plasma membranes (Fig 2). Staining for Env was more frequent on VLPs produced by the MVA vaccines than the DNA vaccine (Fig 2). This is consistent with the gp150 form of Env expressed by the MVA vaccines undergoing higher levels of surface expression and incorporation into virus than the gp160 form expressed by the DNA [13].

### Antigenicity of the directed-lineage Envs

**Fig 3:**
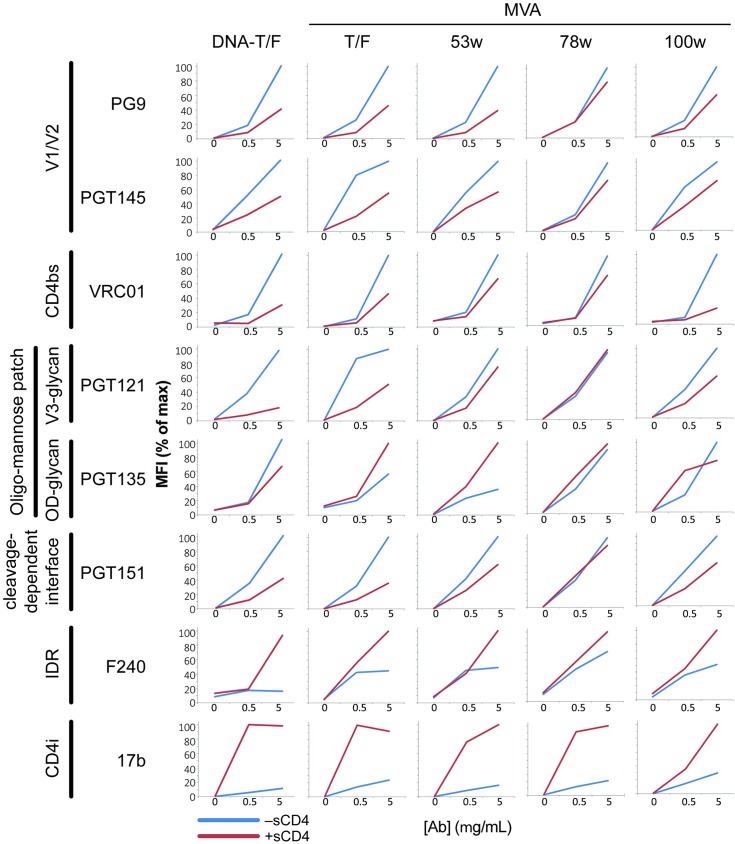
Antigenicity of Env expressed by the 505 vaccines. The vaccines being tested are indicated at the top of the schematic and the recombinant Ab and their specificities on the left side. See Methods for procedures.

Indirect immunofluorescence analysis with Abs, in the presence (red lines) and absence (blue lines) of preincubation with sCD4, revealed that all vaccine-expressed Envs displayed epitopes characteristic of native Env (Fig 3) [25, 26]. Abs used for staining included the PG9 and PGT145 Ab to the V1/V2 quaternary epitope at the tip of the Env trimer, VRC01 to the CD4bs, PGT121 and PGT135 to the V3 and outer domain (OD) glycans of the oligomannose patch, PGT151 to the cleaved interface between gp120 and gp41, F240 to the conserved immunodominant region of gp41 and 17b that recognizes a CD4i-epitope (Fig 3). Except for 17b, all Abs showed good magnitudes of binding in the absence of preincubation with sCD4. 17b gained binding activity following incubation with sCD4. Thus, the Envs required incubation with sCD4 to display the 17b epitope, a prominent signature of CD4 induction.

Whereas sCD4 enhanced binding to the CD4-induced epitope for 17b, it decreased binding to the PG9, PGT145, PGT121 and PGT151 Ab that recognize the native form of Env. This presumably reflected the disruption of these receptor binding epitopes by CD4-induction. The ability of sCD4 to disrupt epitopes of native Env was strongest for the T/F and week 100 Envs (Fig 3).

The 3uM sCD4 did not block the binding of the CD4bs Ab VRC01 to the CH0505 Envs. Because Envs have different susceptibilities to sCD4 binding[27], higher concentrations of sCD4 were tested and 15 μM was found to completely block VRC01 binding (data not shown).

### Immunogenicity testing

To test the ability of the vaccines to raise nAb for the CD4bs, rhesus macaques were immunized with the CH0505 DNA and MVA vaccines as well as gp120 protein (Table 1). The DNA and MVA vaccinations were intended to prime and boost immune responses to the receptor binding form of the CH0505 Env, whereas “late” gp120 protein boosts were intended to enhance primed responses for which the gp120 had cross-reactive epitopes.

**Fig 4:**
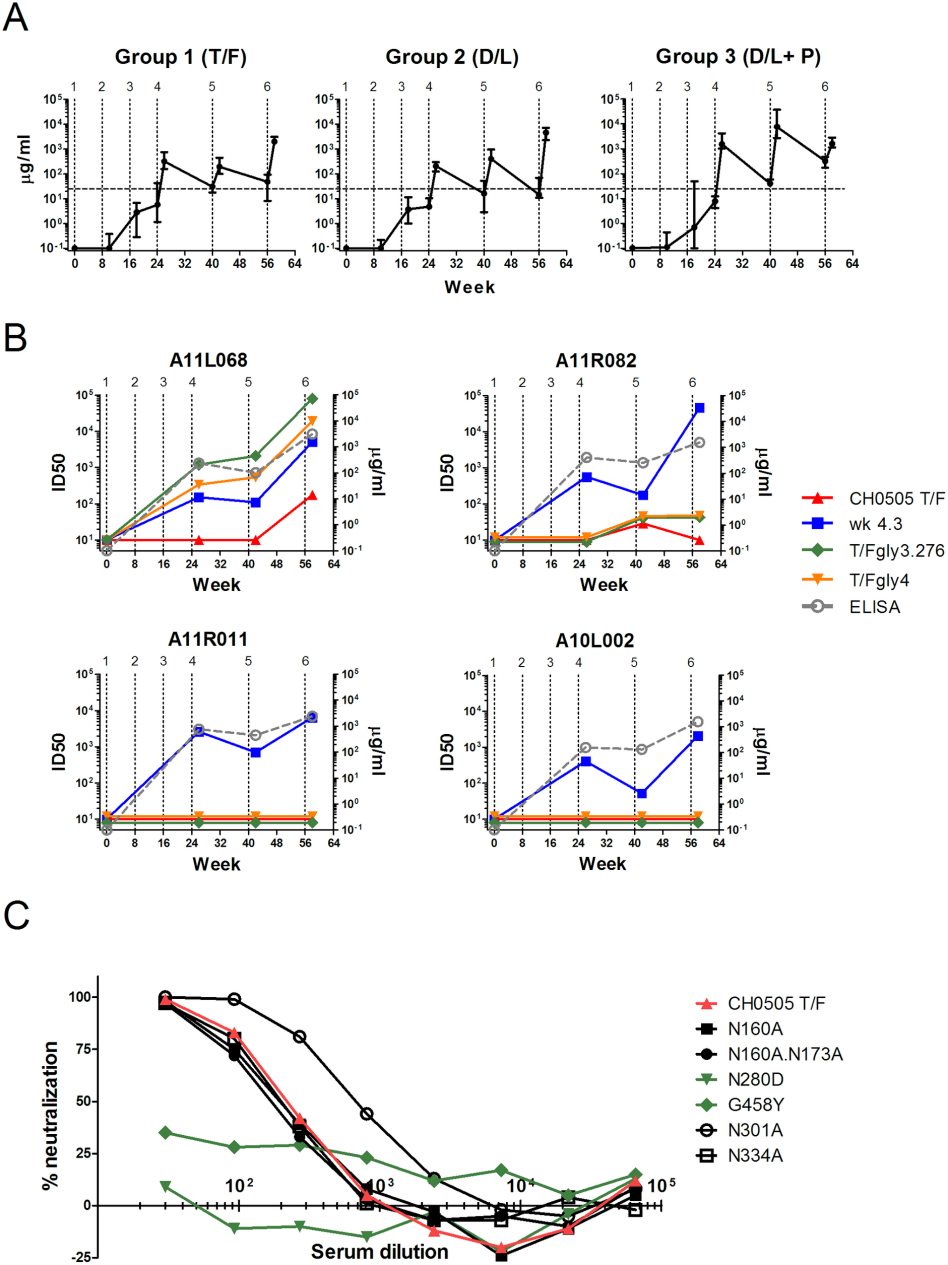
Antibody responses. (A) Patterns of binding Ab for the 3 immunization groups. Vertical dotted lines indicate immunizations. The horizontal dashed line compares troughs across immunizations. (B) Temporal peak titers of neutralizing Ab compared to peak titers of binding Ab (gray dashed line) in Group 1 animals. The patterns for A11R011 and A10L002 are typical for all animals that did not develop nAb to the CD4bs. (C). Mapping of neutralizing activity using mutants that knock out the V1V2 target for bnAb (N160A and N160A.N173A); the CH0505 target for bnAb to the CD4bs (N280D and G458Y); and the V3-glycan target for bnAb (N301A and N334A).

Binding Ab rose with immunizations 1 to 3 following which it increased and contracted with further immunizations (Fig 4A). At peak levels, Ab reached hyperimmune titers as high as 7.7 mg per ml. The troughs had 15 to 300 ug per ml of specific Ab. Overall, the protein boosts increased the titers of responses about 10-fold over those elicited by only MVA. Tests for binding to an antigen representing the CD4bs (RSC3) and RSC3 mutated for its CD4 binding site (RSC3ΔPI) did not reveal Ab for the CD4bs (Data not shown). Titers of neutralizing Ab for a CH0505 Tier 1 virus from week 4.3 of the natural infection, had similar patterns of responses to binding Ab and were overall similar in all animals (for examples see Fig 4B, animals A11R011 and A10L002).

Despite the failure to detect binding Ab for the CD4bs, tests for neutralizing Ab to the CH0505 T/F virus unveiled the presence of neutralizing Ab for the CD4bs in 2 of 4 rhesus receiving only T/F immunizations—one animal with transient low titer tier 2 nAb and one animal with higher titer tier 2 nAb (Fig 4B). This activity did not develop in the 8 macaques receiving T/F plus directed-lineage immunizations. In one macaque (A11R082) low titer neutralizing Ab (29 ID50) was transiently detected after the 5^th^ immunization. Evidence that this neutralization activity was real came from more durable (detected after the 5^th^ and 6^th^ immunizations) and higher titer activity (ID50 of 44–48) against the CH0505TF.gly3.276 and CH505TF.gly4 mutants. These glycan-deleted viruses are highly sensitive to CD4bs antibodies including the precursors of the CD4bs bnAbs isolated from the HIV-infected individual CH0505 (Fig 4B). In the 2^nd^ animal, A11L068, higher titer neutralizing Ab for the 505 T/F virus (ID50 of 152 and 219 in independent assays) appeared post the 6^th^ immunization, which included the 1^st^ gp120 protein boost (Fig 4B). This animal had relatively high titers of neutralizing Ab against the tier 1B CH0505TF.gly3.276 and CH505TF.gly4 mutants by the 4th immunization (ID50 ranging from 334 to 2057). In A11L068 the CD4bs neutralizing activity achieved a plateau of 100% neutralization. The neutralizing Ab were mapped to the CD4bs using CH0505 pseudoviruses with inactivating point mutations in the CD4bs epitope. These mutations knocked out neutralization. In contrast, knockout mutations in the V1V2 glycan site and the V3 glycan site had no effect on neutralization (Fig 4C). Serum neutralizing activity was not enhanced by mutations that increase susceptibility to neutralization by precursors to the VRC-01 lineage of bnAb for the CD4bs (data not shown). The sera did not have neutralizing activity for a 9-virus panel of Tier 2 isolates (data not shown).

## Discussion

Here we show that priming with vectored-immunogens expressing the CH0505 T/F Env on VLPs and boosting with vectored-VLPs plus gp120-T/F protein initiated a B cell lineage with the potential to evolve to heterologous neutralization. The CH0505 T/F Env is a favorable Env for initiating bnAb to the CD4bs because its gp140 form has binding activity (K_d_ = 37.5 nm) for an UCA for bnAb to the CD4bs(V_H_4-59)[9]. We hypothesize that the success of our immunogens could be due to conformationally correct forms of the T/F Env initiating Ab to the CD4bs with the correct angle of approach to permit neutralization of the autologous tier 2 T/F virus. In rhesus A11L068, the T/F gp120 protein appeared to have cross-reactive epitopes capable of boosting the Ab response initiated by the DNA and MVA-T/F VLP expressing vectors.

Up until the 6^th^ immunization, animals in group 1 had received two DNA immunization and 3 MVA immunizations. At the 6^th^ immunization they received MVA-T/F plus gp120-T/F. This protein boost was overall similar in boosting binding Ab and Tier 1 neutralizing responses for all test animals including the two that developed nAb to the CD4bs. For the 2 animals that developed nAb to the CD4bs, the gp120-T/F boosted nAb in one (A11L068) but not the other(A11R082). One possibility is that the two rhesus differed in their germline antibody sequences, where one, but not the other, encoded UCAs that could be gp120-boosted for bnAb lineages such as found in the infection in individual CH0505 (Williams, Verkoczy, Haynes et al, submitted).

Our study provides several precedents for future studies on the use of directed-lineage immunizations to generate bnAb. First, under our current regimen, a sizable number of immunizations may be needed to initiate the lineage from the low frequencies of precursor B cells. Two of 4 macaques in Group 1 that received 6 T/F immunizations, but none of 8 macaques in groups 2 and 3, which received only 3 T/F immunizations, developed nAb to the CD4bs. Thus, initiation of directed-lineage immunizations at the 4^th^ treatment may have been too soon to realize stimulation of a precursor B cell for the CD4bs. In the future, higher vector doses delivered to more than one site (for example each limb) could expedite the initiation of the lineage. Second, we learned that neutralization assays, especially those for highly sensitive viruses from the lineage under study are more useful than ELISAs with target specific antigens (eg RSC3), or Tier 1 neutralizing activity, for detecting initiation of a lineage. Third, we learned that our pulsed immunization took much longer than a natural infection to initiate nAb to the CD4bs (56 weeks as opposed to 14 weeks). Our immunizations were spaced to allow contraction and affinity maturation of responses before reboosting. Temporal contraction needs to be measured to determine if boosts, which presumably have the potential to broaden responses beyond that due to affinity maturation, could be more closely spaced. And last, we learned that high dose protein boosts, at least in the context of co-delivered MVA, elicit hyperimmune responses after more than one delivery. Thus, one may want to reserve such boosts until the end of an immunization regimen, or explore lower doses.

Before the antigenicity studies reported here, we had considered that only gp160 Env would have the structure of native Env and used the gp160 form of Env in the DNA prime to prime a response for native Env. We now appreciate that certain gp150 Envs, such as the CH0505T/F gp150, display Envs that are antigenically indistinguishable from their gp160 form, at least with the panel of Ab that we tested. This is a valuable finding, because expression of gp150 Env supports higher incorporation into VLPs and display on plasma membranes than expression of gp160, which in turn will support higher immunogenicity for Env [15].

The moderate titer neutralizing Ab in A11L068 did not show breadth for other Tier 2 isolates. Going forward, we plan to test whether MVA53C, the Env at the 1^st^ major node for the broadening of nAb responses in patient CH0505, can broaden the Tier 2 nAb for the CD4bs in the responding macaques[9]. We theorize that this broadening may take place relatively rapidly due to the presence of an initiated response with reasonable frequencies of memory B cells for selection and stimulation by the week 53 Env.

## Supporting information

S1 File Binding Ab (ELISA) data–Fig 4A and 4B. (XLSX)

S2 File PAVEG 1232 neutralization data–Fig 4B and data not shown. (XLSX)

S3 File PAVEG 1232 neutralization data—mapping–Fig 4C. (XLSX)

S4 File PAVEG 1232 neutralization data–breadth–data not shown. (XLSX)
